# Orbitally paced phosphogenesis in Mediterranean shallow marine carbonates during the middle Miocene Monterey event

**DOI:** 10.1002/2016GC006299

**Published:** 2016-04-29

**Authors:** Gerald Auer, Christoph A. Hauzenberger, Markus Reuter, Werner E. Piller

**Affiliations:** ^1^ Institute of Earth Sciences, University of Graz, NAWI Graz GeocenterGrazAustria

**Keywords:** middle Miocene, Monterey event, natural gamma radiation, phosphogenesis, orbital forcing, paleoceanography

## Abstract

During the Oligo‐Miocene, major phases of phosphogenesis occurred in the Earth's oceans. However, most phosphate deposits represent condensed or allochthonous hemipelagic deposits, formed by complex physical and chemical enrichment processes, limiting their applicability for the study regarding the temporal pacing of Miocene phosphogenesis. The Oligo‐Miocene Decontra section located on the Maiella Platform (central Apennines, Italy) is a widely continuous carbonate succession deposited in a mostly middle to outer neritic setting. Of particular interest are the well‐winnowed grain to packstones of the middle Miocene Bryozoan Limestone, where occurrences of authigenic phosphate grains coincide with the prominent carbon isotope excursion of the Monterey event. This unique setting allows the analysis of orbital forcing on phosphogenesis, within a bio, chemo, and cyclostratigraphically constrained age‐model. LA‐ICP‐MS analyses revealed a significant enrichment of uranium in the studied authigenic phosphates compared to the surrounding carbonates, allowing natural gamma‐radiation (GR) to be used as a qualitative proxy for autochthonous phosphate content. Time series analyses indicate a strong 405 kyr eccentricity forcing of GR in the Bryozoan Limestone. These results link maxima in the GR record and thus phosphate content to orbitally paced increases in the burial of organic carbon, particularly during the carbon isotope maxima of the Monterey event. Thus, phosphogenesis during the middle Miocene in the Mediterranean was controlled by the 405 kyr eccentricity and its influence on large‐scale paleoproductivity patterns. Rare earth element data were used as a tool to reconstruct the formation conditions of the investigated phosphates, indicating generally oxic formation conditions, which are consistent with microbially mediated phosphogenesis.

## Key Points

- Phosphogenesis during the Monterey event forced by 405 kyr eccentricity controlled paleoproductivity
- Gamma radiation is directly linked to phosphogenesis via U enrichment of authigenic phosphate
- Trace elements show the phosphates formed under microbially mediated and well‐oxygenated conditions

## 1 Introduction

Episodes of globally increased phosphogenesis are widely recognized throughout the Earth's history [*Cook and McElhinny*, 1979; *Föllmi et al*., 1992, 1994, 2005, 2008, 2015; *Föllmi*, 1996; *Jacobs et al*., 1996; *John et al*., 2002]. These events of phosphogenesis correlate well with episodes of strong climatic shifts and carbon isotope excursions, of which the so called Monterey event (∼16.9–13.5 Ma) is a well‐known example during the Miocene [*Woodruff and Savin*, 1991; *Flower and Kennett*, 1994; *Jacobs et al*., 1996; *Abels et al*., 2005; *Holbourn et al*., 2007; *Mourik et al*., 2011; *Diester‐Haass et al*., 2013; *Tian et al*., 2013]. The middle Miocene climate optimum (MMCO) and the subsequent middle Miocene climate transition (MMCT) associated with the Monterey event are of particular interest to the Earths climatic evolution since they represent the beginning shift from the Miocene greenhouse climate to the icehouse climate of the Pleistocene [*Woodruff and Savin*, 1989; *Zachos et al*., 2001a, 2001b, 2008; *Shevenell et al*., 2008; *Tian et al*., 2013]. In combination with decreases in global CO_2_ atmospheric concentrations, the MMCT is often brought into a causal relationship with major changes in the organization of Earth's ocean‐circulation pattern [e.g., *Vincent and Berger*, 1985; *Hamon et al*., 2013]. This global oceanographical reorganization resulted from the formation of North Atlantic Deep Water and the gradual establishment of the modern ocean conveyor belt [*Woodruff and Savin*, 1991, 1989; *Tian et al*., 2009]. A likely cause for this shift is the closure of the Tethyan Seaway as a direct connection between the Indian and the Atlantic Ocean [*Rögl*, 2000; *Steininger and Wessely*, 2000; *Harzhauser et al*., 2002, 2007, 2009; *Reuter et al*., 2009; *Karami et al*., 2011; *Hamon et al*., 2013]. In the Mediterranean, episodes of phosphogenesis coincide with these marked climatic and oceanographic changes [e.g., *Föllmi et al*., 2008]. These widespread occurrences of authigenic phosphate are thus of particular interest in terms of their relationship to the paleoenvironmental and paleoclimatic changes in in the late Oligocene to early late Miocene.

However, as most phosphorite deposits in deeper marine sections of the Mediterranean are highly condensed or were formed through gravitative enrichment processes (e.g., the hemipelagic phosphate deposits of Malta and Sicily) [see *Föllmi et al*., 2008], an exact correlation of authigenic phosphate precipitation and recognized shifts in paleoclimatic conditions has proven difficult, since they lack the needed stratigraphic resolution [*Föllmi*, 1996; *John et al*., 2002; *Stamatakis*, 2004; *Föllmi et al*., 2008, 2015; *Filippelli*, 2011].

In order to fully resolve such episodes of phosphogenesis and to understand underlying geological, climatic, and ecological processes, it is necessary to study the accumulation of authigenic phosphates in sections with sufficiently high sedimentation rates and a low content of reworked phosphate. This also presupposes the need for sections with well‐established correlations to both global chronostratigraphy as well as global climatic records. Furthermore, phosphogenesis needs to be studied with sufficiently high temporal resolution to estimate changes in the amount of phosphate formed over time, in order to accurately correlate them with established palaeoecological and paleoclimatological records. To that end the Decontra section, which is a well‐established shallow‐marine reference section, with a robust orbitally tuned stratigraphic framework [*Mutti and Bernoulli*, 2003; *Reuter et al*., 2013; *Auer et al*., 2015], provides an opportunity to study the relationship between the occurrence of phosphates and global climatic events.

Recent studies found that most occurrences of phosphogenesis in the present day and ancient oceans were directly mediated by microbial activity, irrespective of the marine setting in which they formed [*Föllmi*, 1996; *Mutti and Bernoulli*, 2003; *Hubert et al*., 2005; *Crosby and Bailey*, 2012]. This direct biological control on the creation of one of the primary phosphorous sinks in the Earth's ocean has far‐reaching implications regarding the connection of the global phosphorous with the global carbon cycle as well as local primary productivity variations [*Föllmi*, 1996; *Filippelli*, 1997; *Delaney*, 1998; *Schenau et al*., 2000; *Slomp et al*., 2002; *van der Zee et al*., 2002; *Paytan and McLaughlin*, 2007; *Slomp and Van Cappellen*, 2007]. Using the established stratigraphic framework of the section, it is possible to relate variations in phosphate accumulation within the section to important paleoclimatological and paleoenvironmental changes along the Maiella carbonate ramp and the Mediterranean Sea as a whole.

The present work deals with three questions regarding phosphogenesis in the Decontra section during the MMCO and Monterey event: (1) Determine the phosphate contents on a sufficiently high‐resolution to correlate them with global climatic records; (2) Apply selected rare earth element (REE) proxies to reconstruct the formation history of the phosphates, in order to put them into relation to the already established paleoenvironmental history of the section; (3) Find the dominant uranium‐bearing phase using electron microprobe and LA‐ICP‐MS analyses and understand the proxy‐relationship between these phases and existing gamma log data.

## 2 Setting and Stratigraphy

**Figure 1:**
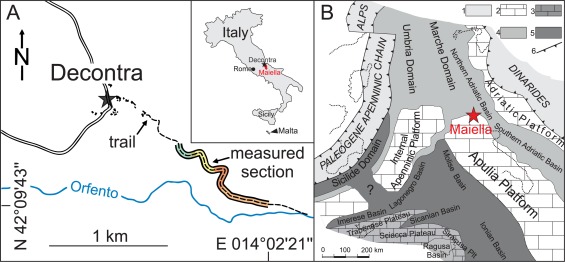
(a) Location Map of the Decontra section, showing its geographical position near the village of Decontra in Central Italy. Colors in the measured section correspond to the informal lithostratigraphic units assigned in figure 2 [after *Reuter et al*., 2013]. (b) Paleogeographical map of the area during the late Oligocene and Miocene [*Patacca et al*., 2008], modified after *Brandano et al*. [2012]. Map shows the isolated nature of the Maiella carbonate system on the northern fringe of the Apulian platform. Major domains depicted are: (1) Paleogene mountain chains; (2) Mesozoic‐Cenozoic carbonate platform domains; (3) pelagic plateaus; (4) basin domains; (5) deep‐water basins floored by oceanic or thinned continental crust; and (6) fronts of orogenic belts.

The late Oligocene to late Miocene Decontra section lies on the northern slope of the Orfento river valley (N 42°09′43.5′′, E 014°02′21.6′′) southeast of the village Decontra on the Maiella mountains in Central Italy (Figure 1). The ∼120 m thick Decontra section is composed of shallow marine carbonates deposited on a gently inclined carbonate ramp along the northern margin of the Apulian Platform [e.g., *Vecsei and Sanders*, 1999]. The section exposes the Bolognano Formation which represents the last depositional unit on the long‐lived Maiella carbonate ramp before the beginning of the Mediterranean salinity crisis [*Crescenti et al*., 1969; *Mutti et al*., 1997; *Vecsei et al*., 1998; *Vecsei and Sanders*, 1999; *Carnevale et al*., 2011].

**Figure 2:**
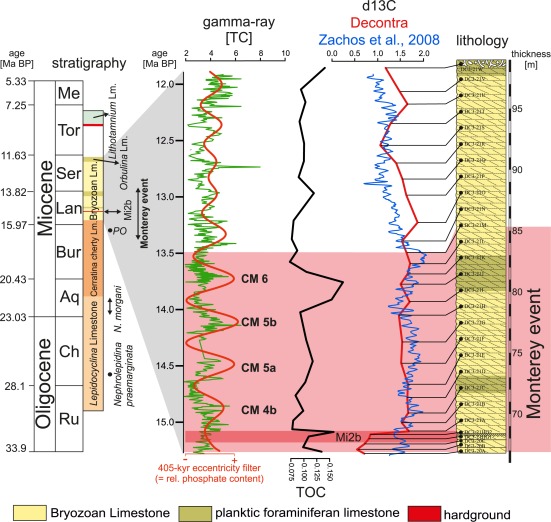
Lithology and orbitally tuned age model shown together with stratigraphic tie‐points of the Decontra section and the Bryozoan Limestone (yellow) with occurrences of intervals rich in planktonic foraminifera (olive) [*Auer et al*., 2015; *Reuter et al*., 2013]. Natural gamma‐radiation is shown as total counts per second, with a Gaussian band‐pass filter applied to the frequency peak corresponding to the orbital 405 kyr eccentricity cycle (see Fig. 3) used as a proxy for long‐term phosphate accumulation in the sediment. Maxima in the gamma‐ray data were correlated to the carbon isotope maxima (CM‐) events [see *Holbourn et al*., 2007], occurring during the Monterey event (highlighted in pink) TOC (black) and carbon isotope (red) curves are plotted adjacent to the gamma‐ray signal. The benthic carbon isotope stack of *Zachos et al*. [2008] is shown for comparison.

Within the section five lithostratigraphic units are described (Figure 2): (1) The 32 m thick *Lepidocyclina* Limestone, which is dominated by larger benthic foraminifera; (2) The Cerratina cherty Limestone—a 35 m thick succession of hemipelagic wackestones to packstones dominated by planktic foraminifers containing chert nodules, phosphatized foraminiferal tests, and sponge spicules. The first occurrence of the planktic foraminifer *Praeorbulina sp*. in the upper part of this unit indicates an age of <16.2 Ma; (3) The 32 m thick Bryozoan Limestone dominated by winnowed bryozoan grainstones with abundant planktonic and benthic foraminifers; (4) The 3 m thick *Orbulina* Limestone with abundant *Orbulina* sp.; (5) The *Lithothamnium* Limestone characterized by abundant red algal fragments overlying the *Orbulina* Limestone with a sharp contact. The base of the *Lithothamnium* Limestone is a 1.5 m thick horizon containing abundant *Heterostegina* fragments [see *Reuter et al*., 2013]. The Decontra section is dated using bio, chemo, and cyclostratigraphy [*Vecsei and Sanders*, 1999; *Carnevale et al*., 2011; *Reuter et al*., 2013; *Auer et al*., 2015]. Regional discrepancies regarding the names of the used informal lithostratigraphic units, still hampers regional correlation of the units [e.g., *Vecsei and Sanders*, 1999; *Cornacchia et al*., 2015]. The lithostratigraphy after *Reuter et al*. [2013] and the current age model of the Decontra section are summarized in Figure 2.

The Decontra section is also well known for occurrences of authigenic phosphate, particularly within the middle Miocene Bryozoan Limestone, where a prominent phosphatic hardground occurs [*Mutti and Bernoulli*, 2003; *Reuter et al*., 2013]. This hardground was extensively studied in the past, and interpreted as an example of early lithification caused by microbially mediated phosphogenesis in a eutrophic shallow marine setting [*Mutti and Bernoulli*, 2003]. While the hardground represents the most prominent example of phosphatisation within the section, the Bryozoan Limestone contains phosphates throughout its well‐sorted 32 m thick low‐angle planar cross‐bedded grainstones dominated by bryozoan and echinoid fragments with frequent occurrences of benthic and planktonic foraminifera in variable quantities. No major observable hiatuses occur throughout the Bryozoan Limestone [*Reuter et al*., 2013]. Planktonic foraminifera‐dominated limestones occur at 72 m, and between 80 and 83 m above the base of the section [*Reuter et al*., 2013; *Auer et al*., 2015]. The sharp contact surface with frequent bioturbation at the top of the so‐called *Orbulina* Limestone, is interpreted as the Ser4/Tor1 sequence boundary [*Reuter et al*., 2013]. Orbital tuning was used to further constrain the age of the Bryozoan Limestone and the overlying *Orbulina* Limestone to ∼15.24–∼11.92 Ma, indicating generally low but continuous sedimentation within this part of the section [*Auer et al*., 2015].

## 3 Methods

The gamma‐ray measurements were carried out in the field in 2012 using a portable “GS‐512” gamma‐ray spectrometer (SatisGeo; measuring time 20 s), and are reported in total counts (TC) [*Reuter et al*., 2013; *Auer et al*., 2015]. Twenty‐seven thin sections (5 × 5 cm) were prepared of the Bryozoan Limestone and used for microfacies analysis and a rough estimation of the phosphate content of the grainstones (Figure 5). Ten selected rock samples were analyzed for their bulk geochemical composition using X‐ray fluorescence spectrometry (XRF) to characterize their overall P_2_O_5_ content and check for impurities in the limestone (mainly the concentrations of K_2_O and MgO as an indicator of glauconite and other clay minerals. XRF analysis was performed at the Institute for Earth Sciences, NAWI Graz, University of Graz with a Bruker Pioneer S4. The ML‐2 standard [*Webb et al*., 2012] (http://www.geoanalyst.org) as well as JLS‐1 (Geological Survey of Japan) were measured as unknowns and reproduced within the reported errors for major components including P_2_O_5_.

P_2_O_5_ was subsequently correlated with the reported gamma‐ray values from the field measurements. Comparison was done by averaging the gamma‐ray measurements in the vicinity of the sampling spot. The results of this comparison together with the correlation coefficient were plotted in a standard cross plot (Figure 4a). To exclude potassium as a significant gamma‐ray source, a similar cross plot was produced for K_2_O and the averaged gamma ray density (Figure 4b).

Based on thin‐section analyses, three representative samples were selected based on the presence of varying amounts of phosphatic grains and phosphatized skeletal material. Polished thick‐sections (∼150–200 µm thickness) of the samples were prepared and documentation of phosphatic grains was performed by backscattered electron (BSE) imaging, using a Jeol Superprobe JXA‐8200 electron microprobe (EMP) at the Eugen F. Stumpfl Electron Microprobe Laboratory, UZAG (University of Leoben, University of Graz, Graz University of Technology). (Figure 6).

Using the BSE images, suitable phosphates were selected for LA‐ICP‐MS analyses. LA‐ICP‐MS analyses were performed at the NAWI Graz Central Lab “Water, Minerals and Rocks” (University of Graz and Graz University of Technology), in order to obtain concentrations for U, Th, and selected rare earth element (REE; see Table 1 for measured elements). A total 29 phosphatic aggregates and nine carbonate reference spots were measured (AP01–AP38; Table1). Spots were measured using a 193 nm laser with a 75 µm spot pulsed at 10 Hz, for very small grains of particular interest (AP7, AP11, AP37) a 50 µm spot was used instead. Dwell time was set at 60 s for each spot, preceded by a 30 s gas blank. Fractionation of elements may have an influence on analytical precision, especially when using nonmatrix‐matched standards. However, as shown by *Williams et al*., [2014] fractionation is generally lower when using a 193 nm wavelength laser system and fractionation of lithophile elements is also generally much smaller compared to siderophile elements, resulting in only small or no fractionation effects for U, Th, and REEs in the analyzed material [*Fryer et al*., 1995].

**Table 1:** Results of the LA‐ICP MS Measurements of the Analyzed Phosphates and Carbonates as well as the Calculated REE Ratios

Sample	Th (ppm)	U (ppm)	La (ppm)	Ce (ppm)	Pr (ppm)	Nd (ppm)	Sm (ppm)	Yb (ppm)	ΣREE (ppm)	Ce/Ce*	Pr/Pr*	Ce Anomaly	La/Sm	La/Yb
Phosphate														
AP01	0.01	***0.63***	28.8	11.9	4.79	21.3	4.52	5.5	104	0.23	1.4	−0.68	0.93	0.39
AP04	1.73	***51.3***	119	74.2	18.4	81.7	18.6	15.2	410	0.36	1.25	−0.49	0.93	0.58
AP05	14.8	***69.8***	596	374	128	565	120	84	2390	0.31	1.35	−0.53	0.72	0.53
AP06	0.24	***57.5***	30.5	18.5	4.93	21.3	3.88	3.7	103	0.34	1.3	−0.51	1.14	0.61
AP07	31.9	***23.6***	1346	668	371	1706	438	163	6252	0.22	1.43	−0.68	0.45	0.61
AP08	2.47	***40.3***	253	127	40.6	181	37	42	879	0.28	1.33	−0.59	1	0.45
AP10	3.4	***37***	237	177	64.7	284	67.9	31.6	1129	0.33	1.38	−0.49	0.51	0.56
AP12	6.49	***132***	602	344	119	494	108	100	2317	0.3	1.42	−0.55	0.81	0.45
AP13	0.57	***29.5***	59.7	31	13.8	65.5	16.2	7.97	257	0.25	1.35	−0.64	0.54	0.55
AP14	6.94	***34.7***	1051	446	215	992	234	173	4233	0.22	1.4	−0.7	0.65	0.45
AP15	19.9	***30.5***	1076	475	241	1161	286	168	4634	0.22	1.36	−0.7	0.55	0.47
AP16	0.31	***35.1***	248	105	43.8	195	47.8	38.2	905	0.23	1.4	−0.67	0.75	0.48
AP18	2.14	***66.6***	375	248	71.8	335	71.4	58.1	1504	0.35	1.25	−0.5	0.77	0.48
AP23	5.33	***14.8***	46.2	23.1	12.7	57	14.5	6.39	213	0.22	1.46	−0.67	0.47	0.53
AP24	5.14	***40.2***	199	85.5	29.7	138	30.7	38	694	0.25	1.31	−0.65	0.95	0.39
AP26	8.97	***60***	408	190	70.4	313	64.1	60.3	1427	0.26	1.37	−0.63	0.93	0.5
AP27	8.85	***43.5***	253	157	61.5	286	66.9	44	1169	0.29	1.34	−0.57	0.55	0.43
AP28	39.9	***58.4***	1497	933	360	1694	419	220	6811	0.29	1.32	−0.56	0.52	0.5
AP29	14.9	***96.1***	1290	874	305	1287	298	158	5402	0.32	1.41	−0.51	0.63	0.6
AP33	3.23	***13.5***	75.2	48.4	21.5	87.3	18.8	7.29	322	0.28	1.53	−0.55	0.58	0.76
AP34	32.4	***51.6***	923	856	259	1073	254	137	4488	0.4	1.38	−0.39	0.53	0.5
AP35	4.11	***57.4***	179	134	46.1	160	33.6	19.6	693	0.34	1.63	−0.45	0.78	0.68
AP37	0.75	***14.2***	86.6	44.3	18.9	77.5	18.2	8.23	320	0.25	1.5	−0.61	0.69	0.78
Carbonate														
AP19	0.11	***0.49***	7.82	3.68	1.78	8.1	1.73	1.55	32.6	0.23	1.41	−0.67	0.66	0.37
AP20	0.02	***0.28***	3.66	1.39	0.56	2.48	0.51	0.63	12.2	0.22	1.4	−0.71	1.05	0.43
AP21	0.27	***0.17***	4.79	1.85	0.96	4.05	0.8	0.57	16.1	0.2	1.52	−0.73	0.88	0.62
AP22	0.69	***0.4***	7.07	3.31	1.33	5.72	1.23	0.83	24	0.25	1.43	−0.64	0.84	0.63
AP25	1.03	***0.38***	7.63	4.34	1.65	7.3	1.55	1.11	29.8	0.28	1.38	−0.58	0.72	0.51
AP30	0.42	***0.23***	8.05	3.66	1.41	6.17	1.25	0.93	26.2	0.25	1.4	−0.64	0.94	0.64
AP31	0.7	***0.38***	6.35	3.34	1.35	5.86	1.2	0.99	24.1	0.26	1.42	−0.6	0.77	0.48
AP32	0.34	***0.18***	7.05	3.42	1.28	5.49	1.2	0.81	23.7	0.26	1.42	−0.62	0.86	0.64
AP38	0.52	***0.99***	6.34	3.13	1.32	5.62	1	0.67	21.7	0.25	1.45	−0.63	0.92	0.7

Standardization of the LA‐ICP‐MS analyses was performed using the NIST standard reference material (SRM) 610 of the National Institute of Standards and Technology, Gaithersburg, MD, USA. Values for the SRMs reported by *Jochum et al*. [2011] were applied for quantification of results. The NIST SRM 612 standard was measured as an unknown to check for accuracy and reproducibility of the LA‐ICP‐MS analyses. Reproducibility of the NIST SRM 612 standard was within <5% relative concentration of U, Th, and the selected REE elements.

While the NIST SRM glasses are not matrix‐matched to the analyzed carbonate and phosphate material, they offer distinct benefits compared to various matrix‐matched standards (i.e., the MACS‐3 carbonate and MAPS‐4 phosphate reference material). Several analytical studies using biogenic carbonates and authigenic apatite found that the analytical advantages offered by the NIST SRM glasses outweigh the disadvantages of standardization using an unmatched matrix, which is largely based on the much better known trace element concentration of the NIST reference glasses compared to other standards [*Jochum et al*., 2012; *Evans and Müller*, 2013; *Caragnano et al*., 2014; *Williams et al*., 2014]. Time‐averaged concentration values of the LA‐ICP‐MS analyses were obtained using GLITTER (ver. 4.0) (Macquarie University, Sydney). Nevertheless, the MAPS‐4 standard was measured as an unknown and reproduced within 1% relative abundance for U, Th, and <5% for the selected REEs, compared to the standard concentrations reported in the Geo‐REM database (http://georem.mpch-mainz.gwdg.de) [*Jochum et al*., 2006].

Electron microprobe (EMP) analyses were subsequently carried out in close proximity to the LA‐ICP‐MS craters, after repolishing the samples. Since the grains are complex and often highly porous mineral aggregates of calcium fluor apatite and calcium carbonate, EMP analysis did not yield results close to the theoretical value of fluor apatite and calcite. Thus, all calculations of analyzed phosphates as well as calcium carbonates utilize a consistent CaO concentration of 55 wt. % as the internal standard value [e.g., *Koenig et al*., 2009].

### 3.1 Data Analysis

Prior to interpretation, the measurements were evaluated for mixed signals of phosphate grains and surrounding carbonate rock based on their major elements (Ca, P, Al, Si). This preevaluation excluded several spots from the subsequent data analyses (AP3, AP09, AP11, AP17, and AP36).

REE concentrations for each spot were transformed into shale‐normalized REE concentrations using the Post Archean Australian Sedimentary Rocks (PAAS) standard [*McLennan*, 1989]. The PAAS standard was chosen for normalization, as it represents a widely used reliable normalization standard for the analysis of REE concentrations in marine environments [*Alibo and Nozaki*, 1999; *Shields and Stille*, 2001; *Haley et al*., 2004; *Garnit et al*., 2012].

PAAS normalized REE concentrations were then used to calculate the Ce anomaly, a useful tool for the characterization of paleoredox conditions [*German and Elderfield*, 1990; *Bau and Dulski*, 1996; *Morad and Felitsyn*, 2001; *Shields and Stille*, 2001; *Garnit et al*., 2012]. *German and Elderfield* [1990] defined the Ce anomaly (Ce/Ce*) as

$$\text{Ce}/{\text{Ce}}^{*}={3(\text{Ce}}_{\text{sample}}/{\text{Ce}}_{\text{shale}})/\left\{{2\;(\text{La}}_{\text{sample}}/{\text{La}}_{\text{shale}})+({\text{Nd}}_{\text{sample}}/{\text{Nd}}_{\text{shale}})\right\}$$

after *De Baar et al*. [1985]. (Table 1 and Figure 7).

Furthermore, the plot Ce/Ce* against Pr/Pr* [*Bau and Dulski*, 1996] can be applied as another way to characterize redox conditions using shale normalized REE concentrations (Table 1 and Figure 8). The Ce/Ce* versus Pr/Pr* plot allows to evaluate the Ce anomaly for possible spurious results caused by anomalous enrichment of La [*Bau and Dulski*, 1996; *Shields and Stille*; 2001; *Garnit et al*., 2012]. For the Ce/Ce* versus Pr/Pr* plot, the anomalies were calculated according to *Bau and Dulski* [1996] as:

$$\text{Ce}/{\text{Ce}}^{*}=({\text{Ce}}_{\text{sample}}/{\text{Ce}}_{\text{shale}})/\left\{0.{5(\text{La}}_{\text{sample}}/{\text{La}}_{\text{shale}})+0.{5(\text{Pr}}_{\text{sample}}/{\text{Pr}}_{\text{shale}})\right\}$$

and

$$\text{Pr}/{\text{Pr}}^{*}=({\text{Pr}}_{\text{sample}}/{\text{Pr}}_{\text{shale}})/\left\{0.{5(\text{Ce}}_{\text{sample}}/{\text{Ce}}_{\text{shale}})+0.{5(\text{Nd}}_{\text{sample}}/{\text{Nd}}_{\text{shale}})\right\}$$

Additionally, the La/Sm ratio was calcluated to account for the possibility that anomalous Nd enrichment may cause a false negative Ce anomaly [*Morad and Felitsyn*, 2001]. A combination of these methods was used to account for possible errors or false positives in either one these methods.

A cross plot of the La/Sm versus La/Yb ratios was used to compare the investigated phosphates to the reported ratio of modern seawater to test for the effect of diagenesis on our samples as proposed by *Reynard et al*. [1999].

**Figure 3:**
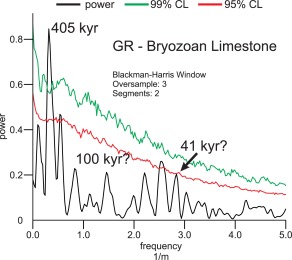
REDFIT power spectrum of the gamma‐ray data from the Bryozoan Limestone [*Auer et al*., 2015]. 99% and 95% Monte‐Carlo corrected confidence intervals are shown as green and red lines, respectively. Frequency‐peaks corresponding to orbital cycles are labeled in kyr, based on sedimentation rate estimates using the stratigraphic model of *Reuter et al*. [2013], *Auer et al*. [2015].

The REDFIT power spectrum for the Bryozoan Limestone shown in Figure 3 was calculated from the GR data by *Auer et al*. [2015] and used for the orbital tuning of the section. The analyses were carried out using the software PAST (version 3.0; http://folk.uio.no/ohammer/past/) [*Hammer et al*., 2001] and are discussed in the aforementioned work.

## 4 Results

### 4.1 Facies and Bulk Sediment Analyses

Thin section analysis of the 27 samples taken within the Bryozoan Limestone reveals well‐winnowed carbonatic grainstones, predominantly composed of bryozoan and echinoid derived skeletal fragments containing a variable amount of planktonic and benthic foraminifera. In the lowermost 2 m of the unit below, the microbially formed phosphatic hardground, notable occurrences of benthic foraminifera (*Amphestigina, Elphidium*, and miliolids) and corallinaceans are common [*Reuter et al*., 2013]. Microbial micrite occurs in the sediment pores just below the hardground [*Mutti and Bernoulli*, 2003; *Reuter et al*., 2013].

Within the Bryozoan Limestone, frequent phosphatisation is observed. Variations in the total amount of phosphatisation are present throughout the investigated interval (Figure 5). The phosphates predominantly occur as diffuse clusters or thin (∼10 µm) filaments throughout the carbonatic sediment. They correspond to the diffuse aggregates and accumulations along grain boundaries observed in thin sections (Figure 5). The phosphate along grain boundaries is mostly oriented parallel to the direction of sedimentation. Phosphatized planktonic and benthic foraminiferal tests, as well as infillings of both bryozoan and echinoid skeletal fragments are also common. Clearly delimited well‐rounded and thus likely transported phosphatic grains are rare. Glauconitic grains, easily identifiably by their distinctive bright green color, are also rare (Figure 5).

Gamma‐ray counts measured in the Bryozoan Limestone unit vary between 1.8 and 7.8 counts per second, with a median of 3.4 and an average of 3.5, the standard deviation is 0.85. Analysis of the gamma‐ray records reveals a clear cyclic variation, which is well‐resolved in the REDFIT power spectrum. The results of the spectral analysis reveal a significant peak at 2.94 m with some additional peaks of lower significance, that were confirmed by Wavelet analysis [*Auer et al*., 2015]. Using the available age model of the section [*Reuter et al*., 2013], the 2.94 m peak closely fits the 405 kyr long eccentricity, showing that the GR‐signal was strongly influenced by orbital parameters. The cyclic pattern matching the 405 kyr eccentricity is well reflected in the Gaussian band‐pass filters applied to the data. Interestingly, the amplitudes of the 2.94 m periodicity show a marked decrease in the middle part of the investigated Bryozoan Limestone unit, corresponding to the end of the Monterey event (Figure 2).

**Figure 4:**
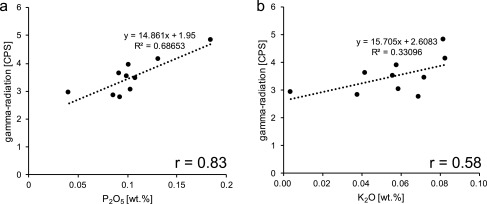
Cross plots showing the relationship between 60 cm average of natural gamma‐radiation field data given in counts per seconds (CPS) and the P_2_O_5_ (a) and K_2_O (b) content of corresponding bulk samples. Correlation coefficient (r) for the respective data sets are shown in the lower right of the plots. High correlation and more inclined regression line indicate a stronger dependency of gamma‐ray intensity on P_2_O_5_ compared to K_2_O. This indicates that U bearing phosphates and not K bearing clays (i.e., glauconite) are the dominant gamma‐ray source.

Results of the XRF analysis show that all samples are very pure carbonates, with only race amounts of SiO_2_, Fe_2_O_3_, and Al_2_O_3_ contained in the sample. The samples have an average P_2_O_5_ content of 0.1 wt. %. Results range from 0.04 to 0.18 wt.% and correlate well with the gamma‐ray intensity obtained in the field (r = 0.83) (Figure 4a). K_2_O is highly accessory with concentrations ranging between ≤0.02 and 0.08 wt.% and is only weakly correlated (r = 0.57), showing that potassium is not a major gamma‐ray source for the sediment (Figure 4b). The correlation between gamma‐ray intensity and Al_2_O_3_, a proxy for phyllosilicates and other terrigenous material, is also weak (r = 0.45; not figured).

### 4.2 Trace Element Analysis

LA‐ICP‐MS and EMP analyses reveal that most investigated phosphatic grains likely represent complex microcrystalline aggregates of phosphate and calcium carbonate, making accurate analysis of the mineral composition difficult. This was further corroborated by the performed electron microprobe analysis of the samples. Nevertheless, matrix standardized results reveal a significant U enrichment of phosphate containing grains in gamma‐ray sources compared to the surrounding purely carbonatic host material. Results of the LA‐ICP‐MS analyses show that the uranium content in the investigated phosphate grains is consistently elevated ranging between 13.5 and 131 ppm, with an average of 48.4 ppm and a median of 43.5 ppm. By comparison, the uranium concentration in the carbonate matrix ranges between 0.17 and 1 ppm, with an average of 0.44 ppm and a median of 0.39 ppm. The standard deviation of the U concentration in the biogenic carbonates is 0.25 ppm. These values result in an average enrichment of roughly 1:111 of uranium in the phosphatic grains compared to the carbonate matrix. Thorium content is comparably low in most analyzed grains, with only three grains showing significantly elevated Th content, in the range of ∼30 ppm (Table 1). Th content in the carbonates is consistently low with ∼0.44 ppm (Table 1). Total REE concentrations (La + Ce + Pr + Nd + Sm + Eu + Gd + Tb + Dy + Ho + Er + Tm + Yb + Lu) in the studied phosphatic grains vary between 104 and 6811 ppm. By comparison, the total REE concentrations of the carbonate range between 4.05 and 33.2 ppm (Table 1).

Calculation of the Ce anomaly following the methods of *German and Elderfield* [1990] and *Morad and Felitsyn* [2001] show a negative anomaly in all samples (Table 1). In the phosphatic grains, the average value of the Ce anomaly is −0.58. The carbonates are comparably similar with an average Ce anomaly of −0.64. Plotting Ce/Ce* against Pr/Pr* enrichment values [*Bau and Dulski*, 1996] further supports the observed negative Ce anomaly as true in all investigated grains. The Ce values can thus be used as an indicator for paleoredox conditions [*Shields and Stille*, 2001; *Garnit et al*., 2012]. The negative Ce anomaly is further confirmed by the La/Sm ratio, which should always be >0.3, in order to rule out that the result an artefact of artificial Nd enrichment [e.g., *Morad and Felitsyn*, 2001]. For our samples, the ratio has consistent values well above the threshold of >0.3 (Table 1).

## 5 Discussion

### 5.1 Natural Gamma‐Radiation as a Proxy for OM Fluxes

Previous studies used a close relationship between the preservation and transport of primary productivity derived organic matter (OM) and both large and small‐scale patterns occur within the gamma‐ray (GR) record of the Decontra section [*Reuter et al*., 2013; *Auer et al*., 2015]. This relationship is of particular importance in the well‐winnowed nearly exclusively carbonatic Bryozoan Limestone. Winnowing by bottom currents effectively removed the small clay and silt‐sized fraction from the sediment leaving a porous grainstone of sand‐sized bryozoan and echinoid fragments, as well as planktonic and benthic foraminifera. Therefore, classical interpretations of variations in the GR record (i.e., content of terrigenous material) are not directly applicable [*Auer et al*., 2015]. The absence of riverine input and a significant amount of aeolian dust within the sediment leads to the need of a conceptual model explaining the observed orbitally paced variations within the Bryozoan Limestone [*Auer et al*., 2015]. The absence of potassium bearing minerals (i.e., clays) in the sediment only leaves uranium and thorium as significant gamma‐ray sources within the sediment. Gamma‐ray probe measurements in the field indicated uranium as the major source of the two. Subsequent laboratory analysis of bulk samples using XRF confirmed these field results and show that K_2_O is only repent in accessory amounts (on average 0.06 wt. %), indicating overall weak contribution of ^40^K to the total gamma ray counts. K_2_O is furthermore only weakly correlated with gamma‐ray intensity (Figure 4b).

The dominance of uranium as GR‐source was further confirmed by the LA‐ICP‐MS data, which indicate consistently higher U than Th concentrations in the phosphate grains, with low concentrations for both elements in the bulk carbonates (Table 1 and supporting information). The intercept of the regression line above zero in the correlation between P_2_O_5_ and total gamma radiation counts (Figure 4a), is thus a results of a minor but overall negligible contribution of K and Th to the overall signal. However, the analytical error of the gamma‐ray spectroscope may also contribute to the offset. Large‐scale trends in the signal should nevertheless be unaffected by stochastic analytical noise.

The comparably long residence time of uranium in the ocean of ∼200–400 kyr renders U concentrations in the ocean water very stable over geological times [*Veeh*, 1967; *Veeh et al*., 1974; *Ku et al*., 1977; *Anderson*, 1982]. The isolated position of the Maiella carbonate ramp during the Miocene (Figure 1) also excludes changes in fluviatile input derived variations of dissolved U concentrations as a cause for the observed variations. Changes in U caused by the deposition of different biogenic carbonates (i.e., varying portions of different skeletal fragments) can also be excluded, since all biogenic carbonates are known to incorporate uranium only during their formation and always in equilibrium with seawater [*Veizer*, 1983; *Ter Kuile and Erez*, 1987, 1988; *Russell et al*., 1994; *Dunk et al*., 2002]. This assumption is also supported by our LA‐ICP‐MS measurements of the carbonate matrix, where even spots placed in close proximity to other mineral grains (i.e., phosphates and iron oxides) exhibit generally low U and Th (∼0.44 ppm) concentrations (Table 1).

Excluding the above mechanisms, variations in POM transport to the ocean floor serve as an effective way to enrich uranium concentrations within the sediment. This assumption is based on the fact, that dissolved uranium ions are preferentially bound to POM in the water column [*Cochran et al*., 1986; *Klinkhammer and Palmer*, 1991; *Spirakis*, 1996; *McManus et al*., 2005]. Together with microbially mediated dissolution of U enriched POM, this creates a primary productivity controlled process of U enrichment within pure carbonate sediments [*Klinkhammer and Palmer*, 1991; *McManus et al*., 2005].

To conclude, the combination of winnowing, U sequestration in the water column by OM and subsequent release of U in the sediment by OM degradation and direct incorporation into microbially formed phosphates [*O'Brien et al*., 1981; *Crosby and Bailey*, 2012] creates an independent primary productivity controlled sink for uranium in the sediment, which circumvents problems with subsequent dissolution and remobilization of U in changing redox conditions [e.g., *Piper and Calvert*, 2009], since these occur contemporary within the sediment water interface, leading to both U and P enrichment, in an environment were microbes are known to be active [*Crosby and Bailey*, 2012] (Figure 9).

This coupling of microbial and redox processes makes uranium a more accurate proxy for relative primary productivity variations in oxic environments, than direct measurements of total organic carbon (TOC). TOC is generally lost in the sedimentary record, as it is quickly dissolved and refracted by biological processes in most settings. This also explains the comparably low organic carbon content of the investigated grainstones (Figure 2). TOC values are nevertheless slightly raised during the Monterey event, and show a prominent peak that can be correlated to CM6 (Figure 2).

### 5.2 Uranium as a Proxy for Relative Phosphate Concentrations

The aforementioned model does, however, not account for the stable mineral phase, binding U within the lithified sediment. Calcium fluor apatite (CFA) is assumed to be the predominant U bearing mineral phase within the sediment [*Auer et al*., 2015]. In light of the existing thin‐section analyses, SEM and EMP BSE micrographs, as well as subsequent LA‐ICP‐MS analysis, we can now confirm this hypothesis. Average U concentrations of the CFA aggregates are ∼48.4 ppm compared to ∼0.44 ppm in carbonates (Table 1). Phosphatic grains thus show a ∼110‐fold uranium enrichment compared to pure carbonate. This analysis is supported by the strong correlation between field gamma‐ray intensity and P_2_O_5_ content of the XRF measurements of discrete rock samples (Figure 4a).

The fact that no other significant U sources occur within the sediment, and U concentrations of the carbonates remained relatively constant (see section 5.1 for discussion) allows the use of variations within the GR record of the Bryozoan Limestone as a proxy for the relative concentration of phosphate within the section. Similarly, a positive correlation between GR records and phosphorous concentration were also reported for phosphate deposits along the Florida platform [*Compton et al*., 1993].

**Figure 5:**
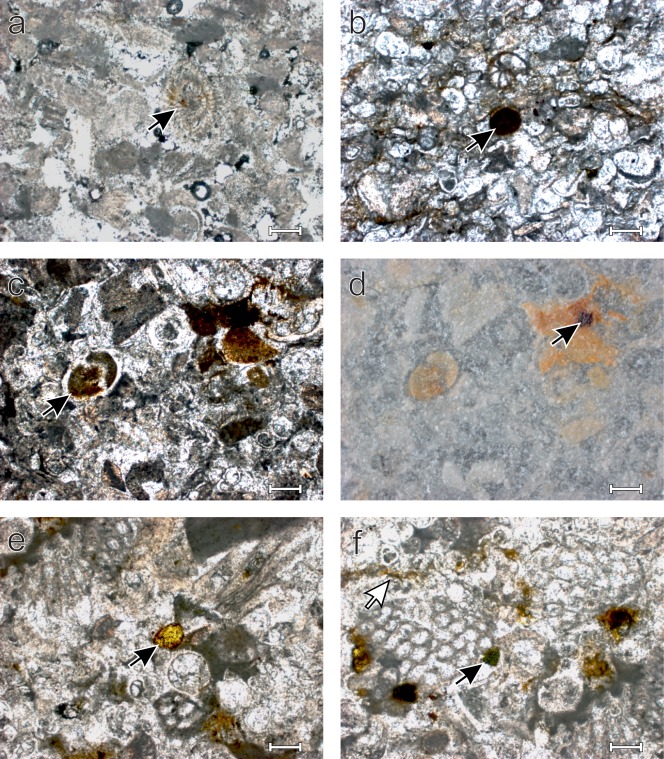
Transmitted (a–c, e, f) and reflected (d) light micrographs of thin sections from the Bryozoan Limestone, Scale bar = 200 µm. (a) Sample DC3‐20B. Arrow shows minor phosphatisation of the chambers of *Amphistegina* sp.; sample has generally low phosphate contents corresponding to the gamma‐ray trough before CM 4b (cf. Figure 2). (b) Diffuse authigenic phosphate coating grains in a sample dominated by planktonic foraminifers (some phosphatized). Sample shows generally high phosphate contents, corresponding to a peak in the gamma‐ray data (Figure 2; DC3‐21J). Black arrow indicates a phosphatized peloidal coprolite surrounded by diffuse phosphate (c) Transmitted light image of a plankton rich bryozoan fragment grainstone; sample DC3‐21C. Phosphates occur as diffuse clusters within the sample; white arrow indicates a phosphatized hyaline foraminiferal test. (d) Corresponding reflected light image to Figure 5c; black arrow indicates iron oxide located in the center of a dense phosphate cluster. (e) Bryozoan fragment and planktonic foraminifers (e.g., *Orbulina* sp.) dominated grainstone with diffuse phosphates associated with microbial micrite. Black arrow: well‐rounded detrital phosphate grain. Sample DC3‐21H. (f) Bryozoan fragment dominated grainstone, with microbial micrite and diffuse authigenic phosphorite in diffuse aggregates that are following grain boundaries (white arrowhead) or are emplaced in micrite; black arrow indicates a reworked glauconite grain; sample DC3‐21H (see Figure 6 for BSE images showing a similar features).

**Figure 6:**
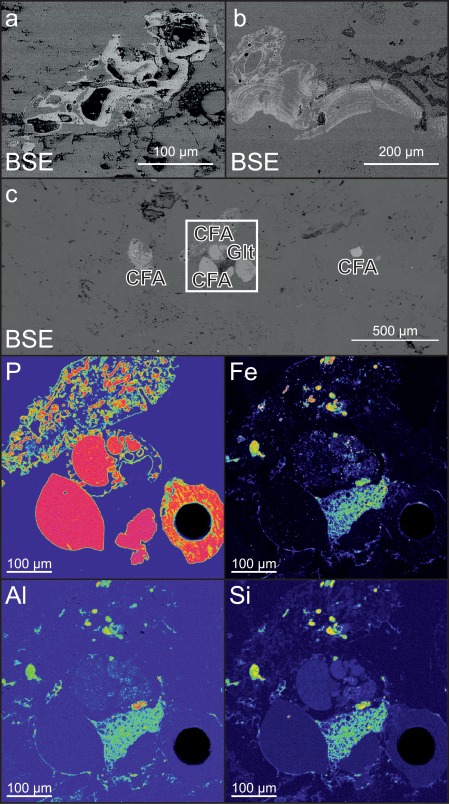
(a) Thick filamentous phosphate (Sample DC3‐21B); (b) fine filamentous phosphate aggregate (Sample DC3‐21H). This phosphate type is associated with the thin diffuse layers shown in Figure 5f. BSE image of a thick section prepared for LA‐ICP‐MS analyses (sample: DC3‐21D; see Figure 2). Light grey areas in the BSE image correspond to phosphate grains (CFA), bright white areas correspond to iron oxides (OX), and darker grey areas correspond to minor occurrences of glauconites (Glt) within the carbonates (medium gray area) of the Bryozoan Limestone. White square in the BSE image represents the area shown in the EPMA elemental distribution maps of Phosphorous (P); note the diffuse authigenic phosphate in upper left associated with a phosphatized benthic foraminiferal test, Iron (Fe) was used to trace iron oxides; together with Aluminum (Al) and Silica (Si) indicates silicates like authigenic glauconite. The black spot represents an ablation crater left by the LA‐ICP‐MS analysis.

Furthermore, the abundant diffuse phosphate observed in thin sections as well as the BSE images indicate a predominantly authigenic nature of the phosphates within the Decontra section, creating a link between phosphogenesis and the GR record (Figures 4, 5, 6). These results offer evidence that variations in GR are a useful tool for the relative estimation of the phosphate content within pure carbonates. Variations in phosphogenesis can consequently be reconstructed in unprecedented high resolution within the Decontra section (Table 1; Figure 2). In combination with the existing orbitally tuned age model [see *Auer et al*., 2015], it becomes possible to reconstruct the processes involved in phosphate formation on the Maiella ramp during the middle Miocene and to relate authigenic phosphate accumulation rates to global paleoclimatological records.

### 5.3 Conceptual Model of Phosphogenesis on the Maiella Ramp

Primary phosphogenesis circumscribes the formation of authigenic phosphate minerals, which predominantly occurs as calcium fluor apatite (CFA) in the marine realm [*Ruttenberg and Berner*, 1993; *Föllmi*, 1996; *van der Zee et al*., 2002; *Filippelli*, 2011]. The processes of phosphogenesis occur in a wide range of settings and, over the past decades, evidence for the constant and widespread occurrence of phosphogenesis on the Earth's ocean floors was found, making the formation of authigenic phosphates a global phenomenon [*Ruttenberg and Berner*, 1993; *Föllmi*, 1996; *Filippelli*, 1997; *van der Zee et al*., 2002; *Föllmi et al*., 2005; *Arning et al*., 2009; *Crosby and Bailey*, 2012]. Current models of CFA formation vary for different settings and are orchestrated by a complex interplay of different processes [e.g., *Föllmi*, 1996]: (1) Precipitation of phosphate through redox‐dependent absorption/desorption processes on Fe‐Mn hydroxides. (2) Formation of phosphates in areas of high primary productivity and high net organic matter accumulation. (3) Seawater‐derived phosphogenesis in settings with reduced sedimentation rates, early lithification, or impermeable substrates. Recent studies furthermore underscored the importance of microbial mats for the occurrence of phosphogenesis in the marine realm [*Arning et al*., 2009; *Filippelli*, 2011; *Crosby and Bailey*, 2012].

The comparably low sedimentation rates for the Bryozoan Limestone (∼9 mm kyr^−1^) [*Auer et al*., 2015] create a setting that was conductive to the formation of microbially precipitated authigenic phosphates (Figure 5) similar to recent examples from the east coast of Australia [*O'Brien et al*., 1981]. Incidentally evidence for this microbial activity was also reported for the prominent hardground ∼2 m above the base of the Bryozoan Limestone [*Mutti and Bernoulli*, 2003], which is bio, chemo, and cyclostratigraphically correlated to the Mi2b glacial event [*Reuter et al*., 2013; *Auer et al*., 2015] (Figure 2). Phosphate accumulation on the Maiella carbonate ramp was consequently heavily influenced by orbitally and thus climatologically controlled variations in primary productivity and relative sea level.

Reduction of sediment accumulation coupled with increased primary productivity during eccentricity minima caused the growth of microbial mats on winnowed sediment grains. At the same time particulate organic matter (POM), the main constituent of marine snow, acts as a transport mechanism for organically bound phosphate to the sediment.

This setting led to a complex interplay of organic matter transport (i.e., marine snow), authigenic/microbial phosphate formation, and uranium incorporation. Release of OM‐derived P in the sediment pore water occurred through degradation of both microbial mats and marine snow through microbial activity within the sediment. Microbes at the sediment water interface used POM as nutrient source, which in turn liberated P and U into a closed biochemically controlled micromilieu at the sediment water interface [*Föllmi*, 1996; *Mutti and Bernoulli*, 2003; *Arning et al*., 2009; *Filippelli*, 2011; *Crosby and Bailey*, 2012] (Figure 9). Here P and U are incorporated into the synchronously precipitating authigenic phosphates. Since the intensity of phosphate precipitation is consequently directly controlled by the amount of OM delivered to the ocean floor, this processes follows large‐scale productivity patterns and preserves the observed orbital trends in the uranium signal, that are only rarely preserved in TOC records, since most of it is lost to this refraction processes (Figures 6a, 6b, and 9).

Thin section and geochemical evidence (see section 5.2) support that phosphogenesis on the Maiella ramp occurred as a microbially mediated process in a dynamic current‐energy‐regime. Microbial activity in turn was largely guided by variable nutrient supply caused by changes in organic matter flux from the ocean surface via primary productivity controlled marine snow (Figure 9). This process is commonly suggested as the cause of nutrient limitation within aphotic microbial communities [e.g., *Davey and O'toole*, 2000].High current energies and the coarse well‐winnowed sediment further precluded extensive redox cycling of dissolved P as well as Fe and Mn species, since sufficient anoxia for iron hydroxide reduction only formed in deeper parts of the sediment where no direct exchange with the ocean water was possible. This largely disabled the “redox pump” as a process for non‐OM‐derived P enrichment within the sediment of the Decontra section (Figure 9) [*Föllmi*, 1996; *Schenau et al*., 2000; *van der Zee et al*., 2002; *Paytan and McLaughlin*, 2007; *Filippelli*, 2011; *Crosby and Bailey*, 2012]. The absence of any significant anoxia in the water column or within the sediment, is further supported by the absence of sulfides in the Bryozoan Limestone [*Reuter et al*., 2013; *Auer et al*., 2015].

**Figure 7:**
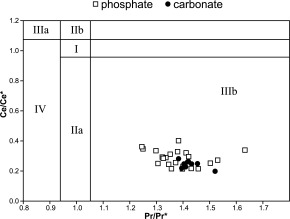
Ce/Ce* versus Pr/Pr* plot after *Bau and Dulski* [1996]. *Garnit et al*. [2012] described the fields of the plot as follows: Field I: no anomaly; Field IIa: positive La anomaly causes apparent negative Ce anomaly; Field IIb: negative La anomaly causes apparent positive Ce anomaly; Field IIIa: real positive Ce anomaly; Field IIIb: real negative Ce anomaly; Field IV: positive La anomaly disguises positive Ce anomaly. Using this interpretation, all analyzed phosphates as well as biogenic carbonates plot in the IIIb field, indicating the presence of a real negative Ce anomaly, indicative of oxic formation conditions.

**Figure 8:**
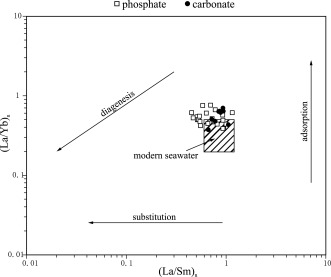
(La/Yb)_n_ versus (La/Sm)_n_ ratio plot of both phosphate and carbonate reported in the diagram proposed by *Reynard et al*. [1999] and redrawn after *Garnit et al*. [2012]. Plot indicates that while all analyzed samples are still broadly similar to modern day seawater (note that some samples of both phosphate and carbonate plot within the “modern seawater” field), both adsorption and substitution processes affected the investigated samples to a minor degree. This is in agreement with the current interpretation, indicating early diagenetic formation (i.e., authigenic) or biogenic formation as well as prolonged exposure to seawater in a high current‐energy deposition system with generally low sedimentation rates.

**Figure 9:**
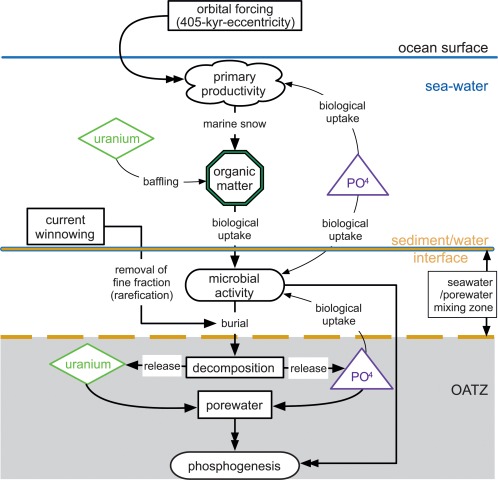
Box model of the processes involved leading to phosphogenesis on the Maiella carbonate ramp. Orbitally paced primary productivity causes increased marine snow. The sinking organic matter within the marine snow takes up uranium from the seawater and transports it to the ocean floor. At the ocean floor, OM acts as the limiting factor for the establishment of microbial communities. Microbial communities also protect the organic matter from winnowing by current activity. Phosphorous is concentrated in the OM and is also taken up directly from the ocean water by the microbial communities at the ocean floor. Decomposition of both primary and microbial mat derived organic matter within the sediment provides both phosphorous and uranium, which is then incorporated into carbonate fluor apatite during primary phosphogenesis within the oxic anoxic transition zone (OATZ) in the sediment.

Redox cycling only occurred in a limited area at the oxic/anoxic transition zone (OATZ) within in the sediment, without significant mixing or enrichment from the overlying ocean water and in conjunction with the activity of magnetotactic bacteria [*Aissaoui et al*., 1990; *McNeill*, 1990; *Frankel and Bazylinski*, 1994; *Fortin and Langley*, 2005; *Maloof et al*., 2007; *Kopp and Kirschvink*, 2008]. This is supported by the investigated REE signatures of the phosphates as well as the biogenic carbonates, which both point towards well‐oxygenated formation conditions (Table 1 and Figures 7, 8, 9).

REE signatures show a negative Ce anomaly in both the carbonate matrix and all investigated phosphatic grains using the Ce/Ce* versus Pr/Pr* plot [*Bau and Dulski*, 1996]. The plot is supported by the calculated Ce anomaly [*German and Elderfield*, 1990] (Figure 7; Table 1). Combined with the available REE signatures of modern pore waters, as well as biogenic, authigenic, and detrital phosphates from the literature indicates that all investigated phosphates were formed in a well‐oxygenated environment [*German and Elderfield*, 1990; *Bau and Dulski*, 1996; *Alibo and Nozaki*, 1999; *Morad and Felitsyn*, 2001; *Haley et al*., 2004; *Shields and Stille*, 2001; *Garnit et al*., 2012; *Emsbo et al*., 2015]. Furthermore, the La/Sm and La/Yb ratios from our data plot remarkably close to or within the reported values for modern seawater (Figure 8).

A slight trend toward more negative La/Sm values as well as enriched La/Yb values occurs in our data. This suggests that both phosphates and carbonates in the Decontra section were affected by substitution processes as well as adsorption processes during early diagenesis [*Reynard et al*., 1999; *Shields and Stille*, 2001; *Garnit et al*., 2012]. In general, however, our results indicate that both carbonates and phosphates were only slightly affected by early diagenesis and that any related alteration most likely occurred at the same time after final deposition. Interestingly Quaternary fish debris shows similar values to the investigated phosphates of the Bryozoan Limestone [see *Reynard et al*., 1999]. Although some contribution of reworked phosphate grains is likely, our results show that all measured phosphates formed in oxic conditions in connection with microbial activity and that their Uranium signal is not a product of subsequent alteration during diagenesis.

This creates a good explanation for the largely in‐phase variability of both the magnetic susceptibility and GR records of the Decontra section [*Auer et al*., 2015], since the formation of microbially derived iron oxides and phosphates predominantly occurred during organic matter decomposition at a microbially controlled OATZ within the sediment. Combining these assumptions with our results allows the construction of a simple box model for the processes involved in phosphogenesis on the Maiella ramp (Figure 9).

### 5.4 Orbitally Paced Phosphogenesis and Its Implications for the Global Phosphorous Cycle

Economically viable phosphorites are generally deposited as lag deposits or hardgrounds formed during episodes of nondeposition or even active sediment removal. Thus, virtually all global phosphorite deposits are generally unrelated to primary P accumulation within the sediment and subsequent phosphogenesis [*Filippelli*, 2011]. Therefore, the Decontra section represents a unique opportunity since the formation history of shallow marine phosphates can be analyzed in the context of an orbitally tuned age model. While the continued occurrence of phosphogenesis throughout the Bryozoan Limestone points to long‐term eutrophic conditions [*Mutti and Bernoulli*, 2003; *Reuter et al*., 2013], the distinct long‐eccentricity forcing until ∼13.5 Myr, indicates a strong orbital control on the phosphate formation. This directly links the accumulation of phosphate on the Maiella platform to variations in primary productivity during the Monterey event (Figures 2, 3, and 9). By extent, the variations in phosphorous accumulation can also be linked to occurring patterns in the global carbon cycle, which are well reflected by the global carbon isotope record [*Vincent and Berger*, 1985; *Jacobs et al*., 1996; *Abels et al*., 2005; *Mourik et al*., 2011; *Holbourn et al*., 2015]. The subsequent breakdown in the 405 kyr eccentricity signal after ∼13.5 Ma points toward a reorganization of ecological conditions controlling primary productivity at the end of the Monterey event, which directly relates to changes in net phosphate accumulation (Figure 2). Thus the marked shift in the preservation of the 405 kyr cyclicity and a general increasing trend in the GR signal (and thus phosphate accumulation) may point to drastically different primary productivity patterns during the early stages of the middle Miocene climate transition and thus a major reorganization of the marine phosphorus and carbon cycles. This proposed reorganization in current patterns coincide with the closure of the Tethyan seaway and Antarctic ice sheet expansion during the MMCT [*J. C. Zachos et al*., 2001a; *J. Zachos et al*. 2001a; *Harzhauser et al*., 2007, 2009], and is thus an expression of major paleogeographical and climatological changes during this time.

Our results indicate that the well‐known link between the carbon and phosphorous cycle [*Föllmi*, 1996; *Filippelli*, 1997; *van der Zee et al*., 2002; *Filippelli*, 2011] can now be extended to orbitally driven variations in net phosphorous burial in shallow marine settings. Changes in this forcing appear to be caused by significant global changes, such as the closure of the Tethyan seaway and the Antarctic ice sheet expansion during the MMCT, masking the orbitally paced pattern, which dominated during the Monterey event [*Holbourn et al*., 2015].

Our results provide insights into the pacing and formation requirements of the widespread middle Miocene shallow marine phosphorite deposits [*Compton et al*., 1993; *Föllmi*, 1996; *Jacobs et al*., 1996; *John et al*., 2002; *Föllmi et al*., 2008], and link them to the eccentricity forcing, global productivity, and climate cycles during the Monterey event for the first time. The orbital pacing of phosphogenesis shows that phosphorous accumulation is coupled to known pacing of primary productivity and organic carbon burial, which are proposed as the underlying causes of the Monterey event and the CM‐events [see *Holbourn et al*., 2007; *Diester‐Haass et al*., 2013; *Holbourn et al*., 2015].

Our model is thus in accordance with the hypothesis that variations in ocean circulation are the cause of the 405 kyr eccentricity‐forcing observed in the δ^13^C record during the Monterey event [*Holbourn et al*., 2007; *Diester‐Haass et al*., 2013; *Holbourn et al*., 2015]. Increased ocean circulation supplied nutrients to shallow marine environments, where stronger coastal and continental upwelling caused increases in primary productivity and thus phosphorous burial along the oxic/anoxic transition zone within the sediment. The removal of high amounts of bioavailable phosphate during the Monterey‐event may thus have played a limiting role on primary productivity, which in turn acted as the limiting factor for the carbon isotope excursion during the Monterey event, preventing further increase. Furthermore, the significantly increased phosphorous burial during the CM‐events may have contributed to the pronounced carbon isotope cyclicity, since the very limited phosphate supply following its increased removal during the CM‐events drastically reduced primary productivity afterwards. This feedback‐loop may thus explain the pronounced 405 kyr cyclicity in the carbon isotope record and provides an important clue for the controlling factors of the Monterey event, which to date are still not fully understood [*Diester‐Haass et al*., 2013; *Holbourn et al*., 2015].

## 6 Conclusions

The present study uses LA‐ICP‐MS data of carbonate grainstone as well as authigenic phosphate to characterize microbially mediated phosphogenesis in the middle Miocene Bryozoan Limestone of the Decontra section in the Maiella mountain range (Central Italy). Our results show that: (1) primary microbially controlled phosphogenesis persisted throughout the Bryozoan Limestone in accordance with previous studies [*Mutti and Bernoulli*, 2003; *Reuter et al*., 2013]. (2) LA‐ICP‐MS analyses confirm that phosphate is the primary carrier of uranium within the sediment. (3) The current‐dominated, high‐energy deposystem of the Bryozoan Limestone removed all other gamma‐ray sources from the sediment, leaving phosphates as the primary source of gamma‐radiation within the Bryozoan Limestone. This allows the GR data to be used as a high‐resolution proxy for the relative phosphate content of the sediment. (4) The strong 405 kyr orbital control on the GR record found in previous studies [*Auer et al*., 2015] can now be linked to the phosphate content of the section. This link provides new insights into primary productivity and organic carbon burial cycles during the Monterey event. We show that increased calcium fluor apatite burial occurred in shallow marine carbonates during the carbon isotope maxima, which links Miocene phosphogenesis to eccentricity forcing. Phosphogenesis during the middle Miocene in the Mediterranean was thus controlled by the 405 kyr eccentricity and its influence on large‐scale paleoproductivity patterns. (5) Significant changes occurred in the phosphate accumulation patterns at the end of the Monterey event, which can be related to changes in ocean circulation patterns caused by the closure of the Tethyan seaway and the onset in Antarctic ice sheet buildup. (6) Shale normalized rare earth element analysis of the investigated phosphates using the Ce anomaly and La/Sm ratio in combination with the Ce/Ce* versus Pr/Pr* and La/Sm versus La/Yb ratio plot proved useful in the reconstruction of their formation conditions, supporting their microbially mediated origin in a eutrophic and well‐oxygenated setting.

## Supporting information

Supporting Information S1

Table S1

## References

[ggge21000-bib-0001] Abels, H. A. , F. J. Hilgen , W. Krijgsman , R. W. Kruk , I. Raffi , E. Turco , and W. J. Zachariasse (2005), Long‐period orbital control on middle Miocene global cooling: Integrated stratigraphy and astronomical tuning of the Blue Clay Formation on Malta, Paleoceanography, 20, PA4012, doi:10.1029/2004PA001129.

[ggge21000-bib-0002] Aissaoui, D. M. , D. F. McNeil , and J. L. Kirschvink (1990), Magnetostratigraphic dating of shallow‐water carbonates from Mururoa atoll, French Polynesia: Implications for global eustasy, Earth Planet. Sci. Lett., 97(1–2), 102–112, doi:10.1016/0012-821X(90)90102-4.

[ggge21000-bib-0003] Alibo, D. S. , and Y. Nozaki (1999), Rare earth elements in seawater: Particle association, shale‐normalization, and Ce oxidation, Geochim. Cosmochim. Acta, 63(3–4), 363–372, doi:10.1016/S0016-7037(98)00279-8.

[ggge21000-bib-0004] Anderson, R. F. (1982), Concentration, vertical flux, and remineralization of particulate uranium in seawater, Geochim. Cosmochim. Acta, 46(7), 1293–1299, doi:10.1016/0016-7037(82)90013-8.

[ggge21000-bib-0005] Arning, E. T. , D. Birgel , B. Brunner , and J. Peckmann (2009), Bacterial formation of phosphatic laminites off Peru, Geobiology, 7(3), 295–307, doi:10.1111/j.1472-4669.2009.00197.x. 19476504 10.1111/j.1472-4669.2009.00197.x

[ggge21000-bib-0006] Auer, G. , W. E. Piller , M. Reuter , and M. Harzhauser (2015), Correlating carbon and oxygen isotope events in early to middle Miocene shallow marine carbonates in the Mediterranean region using orbitally tuned chemostratigraphy and lithostratigraphy, Paleoceanography, 30, 332–352, doi:10.1002/2014PA002716. 10.1002/2014PA002716 PMC4974900 27546980

[ggge21000-bib-0007] Bau, M. , and P. Dulski (1996), Distribution of yttrium and rare‐earth elements in the Penge and Kuruman iron‐formations, Transvaal Supergroup, South Africa, Precambrian Res., 79(1–2), 37–55, doi:10.1016/0301-9268(95)00087-9.

[ggge21000-bib-0008] Brandano, M. , L. Lipparini , V. Campagnoni , and L. Tomassetti (2012), Downslope‐migrating large dunes in the Chattian carbonate ramp of the Majella Mountains (Central Apennines, Italy), Sediment. Geol., 255–256(0), 29–41, doi:10.1016/j.sedgeo.2012.02.002.

[ggge21000-bib-0009] Caragnano, A. , D. Basso , D. E. Jacob , D. Storz , G. Rodondi , F. Benzoni , and E. Dutrieux (2014), The coralline red alga Lithophyllum kotschyanum f. affine as proxy of climate variability in the Yemen coast, Gulf of Aden (NW Indian Ocean), Geochim. Cosmochim. Acta, 124, 1–17.

[ggge21000-bib-0010] Carnevale, G. , E. Patacca , and P. Scandone (2011), Field Guide to the Post‐Conference Excursions (Scontrone, Palena and Montagna della Majella), pp. 1–98, R.C.M.N.S. Interim Colloquium, Scontrone, Italy.

[ggge21000-bib-0011] Cochran, J. K. , A. E. Carey , E. R. Sholkovitz , and L. D. Surprenant (1986), The geochemistry of uranium and thorium in coastal marine sediments and sediment pore waters, Geochim. Cosmochim. Acta, 50(5), 663–680.

[ggge21000-bib-0012] Compton, J. S. , D. A. Hodell , J. R. Garrido , and D. J. Mallinson (1993), Origin and age of phosphorite from the south‐central Florida Platform: Relation of phosphogenesis to sea‐level fluctuations and δ13C excursions, Geochim. Cosmochim. Acta, 57(1), 131–146, doi:10.1016/0016-7037(93)90474-B.

[ggge21000-bib-0013] Cook, P. J. , M. W. McElhinny (1979), A reevaluation of the spatial and temporal distribution of sedimentary phosphate deposits in the light of plate tectonics, Econ. Geol., 74(2), 315–330, doi: 10.2113/gsecongeo.74.2.315.

[ggge21000-bib-0014] Cornacchia, I. , M. Brandano , I. Raffi , and L. Tomassetti (2015), Integrated stratigraphy of the Bolognano Formation (Majella, Central Apennines): What's new?, vol. XII, pp. 1–4, XII Congresso GeoSed‐Sezione Geologia del Sedimentario SGI. Cagliari.

[ggge21000-bib-0015] Crescenti, U. , A. Crostella , G. Donzelli , and G. Raffi (1969), Stratigrafia della serie calcarea dal Lias al Miocene nella regione marchigiano‐abruzzese, Mem. Soc. Geol. It., 8, 343–420.

[ggge21000-bib-0016] Crosby, C. H. , and J. V. Bailey (2012), The role of microbes in the formation of modern and ancient phosphatic mineral deposits, Front. Microbiol., 3(241), 1–7, doi:10.3389/fmicb.2012.00241. 22783245 10.3389/fmicb.2012.00241 PMC3389779

[ggge21000-bib-0017] Davey, M. E. , and G. A. O'toole (2000), Microbial biofilms: From ecology to molecular genetics, Microbiol. Mol. Biol. Rev., 64(4), 847–867, doi:10.1128/MMBR.64.4.847-867.2000. 11104821 10.1128/mmbr.64.4.847-867.2000 PMC99016

[ggge21000-bib-0018] De Baar, H. J. W. , M. P. Bacon , P. G. Brewer , and K. W. Bruland (1985), Rare earth elements in the Pacific and Atlantic Oceans, Geochim. Cosmochim. Acta, 49(9), 1943–1959, doi:10.1016/0016-7037(85)90089-4.

[ggge21000-bib-0019] Delaney, M. L. (1998), Phosphorus accumulation in marine sediments and the oceanic phosphorus cycle, Global Biogeochem. Cycles, 12(4), 563–572, doi:10.1029/98GB02263.

[ggge21000-bib-0020] Diester‐Haass, L. , K. Billups , I. Jacquemin , K. C. Emeis , V. Lefebvre , and L. François (2013), Paleoproductivity during the middle Miocene carbon isotope events: A data‐model approach, Paleoceanography, 28, 334–346, doi:10.1002/palo.20033.

[ggge21000-bib-0021] Dunk, R. M. , R. A. Mills , and W. J. Jenkins (2002), A reevaluation of the oceanic uranium budget for the Holocene, Chem. Geol., 190(1–4), 45–67, doi:10.1016/S0009-2541(02)00110-9.

[ggge21000-bib-0022] Emsbo, P. , P. I. McLaughlin , G. N. Breit , E. A. du Bray , and A. E. Koenig (2015), Rare earth elements in sedimentary phosphate deposits: Solution to the global REE crisis? Gondwana Res., 27(2), 776–785, doi:10.1016/j.gr.2014.10.008.

[ggge21000-bib-0023] Evans, D. , and W. Müller (2013), LA‐ICPMS elemental imaging of complex discontinuous carbonates: An example using large benthic foraminifera, J. Anal. At. Spectrom., 28(7), 961–1132, doi:10.1039/C3JA50053E.

[ggge21000-bib-0024] Filippelli, G. M. (1997), Controls on phosphorus concentration and accumulation in oceanic sediments, Mar. Geol., 139(1–4), 231–240, doi:10.1016/S0025-3227(96)00113-2.

[ggge21000-bib-0025] Filippelli, G. M. (2011), Phosphate rock formation and marine phosphorus geochemistry: The deep time perspective, Phosphorus Cycle, 84(6), 759–766, doi:10.1016/j.chemosphere.2011.02.019. 10.1016/j.chemosphere.2011.02.019 21376366

[ggge21000-bib-0026] Flower, B. P. , and J. P. Kennett (1994), The middle Miocene climatic transition: East Antarctic ice sheet development, deep ocean circulation and global carbon cycling, Palaeogeogr. Palaeoclimatol. Palaeoecol., 108(3–4), 537–555, doi:10.1016/0031-0182(94)90251-8.

[ggge21000-bib-0027] Fortin, D. , and S. Langley (2005), Formation and occurrence of biogenic iron‐rich minerals, Earth Sci. Rev., 72(1–2), 1–19, doi:10.1016/j.earscirev.2005.03.002.

[ggge21000-bib-0028] Föllmi, K. B. (1996), The phosphorus cycle, phosphogenesis and marine phosphate‐rich deposits, Earth Sci. Rev., 40(1–2), 55–124, doi:10.1016/0012-8252(95)00049-6.

[ggge21000-bib-0029] Föllmi, K. B. , R. E. Garrison , P. C. Ramirez , F. Zambrano‐Ortiz , W. J. Kennedy , and B. L. Lehner (1992), Cyclic phosphate‐rich successions in the upper Cretaceous of Colombia, Palaeogeogr. Palaeoclimatol. Palaeoecol., 93(3–4), 151–182, doi:10.1016/0031-0182(92)90095-M.

[ggge21000-bib-0030] Föllmi, K. B. , H. Weissert , M. Bisping , and H. Funk (1994), Phosphogenesis, carbon‐isotope stratigraphy, and carbonate‐platform evolution along the Lower Cretaceous northern Tethyan margin, Geol. Soc. Am. Bull., 106(6), 729–746, doi:10.1130/0016-7606(1994)106<0729:PCISAC>2.3.CO;2.

[ggge21000-bib-0031] Föllmi, K. B. , C. Badertscher , E. de Kaenel , P. Stille , C. M. John , T. Adatte , and P. Steinmann (2005), Phosphogenesis and organic‐carbon preservation in the Miocene Monterey Formation at Naples Beach, California: The Monterey hypothesis revisited, Geol. Soc. Am. Bull., 117(5), 589–619, doi:10.1130/B25524.1.

[ggge21000-bib-0032] Föllmi, K. B. , B. Gertsch , J. P. Renevey , E. De Kaenel , and P. Stille (2008), Stratigraphy and sedimentology of phosphate‐rich sediments in Malta and south‐eastern Sicily (latest Oligocene to early Late Miocene), Sedimentology, 55(4), 1029–1051.

[ggge21000-bib-0033] Föllmi, K. B. , H. Hofmann , M. Chiaradia , E. de Kaenel , G. Frijia , and M. Parente (2015), Miocene phosphate‐rich sediments in Salento (southern Italy), Sediment. Geol., 327 IS *‐*, 55–71, doi:10.1016/j.sedgeo.2015.07.009.

[ggge21000-bib-0034] Frankel, R. B. , and D. A. Bazylinski (1994), Magnetotaxis and magnetic particles in bacteria, Hyperfine Interact., 90(1), 135–142–142, doi:10.1007/BF02069123.

[ggge21000-bib-0035] Fryer, B. J. , S. E. Jackson , and H. P. Longerich (1995), The design, operation and role of the laser‐ablation microprobe coupled with an inductively coupled plasma‐mass spectrometer (LAM‐ICP‐MS) in the earth sciences, Can. Mineral., 33, 303–312.

[ggge21000-bib-0036] Garnit, H. , S. Bouhlel , D. Barca , and C. Chtara (2012), Application of LA‐ICP‐MS to sedimentary phosphatic particles from Tunisian phosphorite deposits: Insights from trace elements and REE into paleo‐depositional environments, Chem. Erde Geochem, 72(2), 127–139, doi:10.1016/j.chemer.2012.02.001.

[ggge21000-bib-0037] German, C. R. , and H. Elderfield (1990), Application of the Ce anomaly as a paleoredox indicator: The ground rules, Paleoceanography, 5(5), 823–833, doi:10.1029/PA005i005p00823.

[ggge21000-bib-0038] Haley, B. A. , G. P. Klinkhammer , and J. McManus (2004), Rare earth elements in pore waters of marine sediments, Geochim. Cosmochim. Acta, 68(6), 1265–1279, doi:10.1016/j.gca.2003.09.012.

[ggge21000-bib-0039] Hammer, Ø. , D. A. T. Harper , and P. D. Ryan (2001), *Past: Paleontological Statistics Software Package for Education and Data Analysis, Palaeontologia Electronica*, vol. 4, 1(4), 9.

[ggge21000-bib-0040] Hamon, N. , P. Sepulchre , V. Lefebvre , and G. Ramstein (2013), The role of eastern Tethys seaway closure in the Middle Miocene Climatic Transition (ca. 14 Ma), Clim. Past, 9(6), 2687–2702, doi:10.5194/cp-9-2687-2013.

[ggge21000-bib-0041] Harzhauser, M. , W. E. Piller , and F. F. Steininger (2002), Circum‐Mediterranean Oligo–Miocene biogeographic evolution: The gastropods' point of view, Palaeogeogr. Palaeoclimatol. Palaeoecol., 183(1–2), 103–133.

[ggge21000-bib-0042] Harzhauser, M. , A. Kroh , O. Mandic , W. E. Piller , U. Göhlich , M. Reuter , and B. Berning (2007), Biogeographic responses to geodynamics: A key study all around the Oligo–Miocene Tethyan Seaway, Zool. Anz., 246(4), 241–256, doi:10.1016/j.jcz.2007.05.001.

[ggge21000-bib-0043] Harzhauser, M. , M. Reuter , W. Piller , B. Berning , A. Kroh , and O. Mandic (2009), Oligocene and Early Miocene gastropods from Kutch (NW India) document an early biogeographic switch from Western Tethys to Indo‐Pacific, Palaeont. Z., 83(3), 333–372, doi:10.1007/s12542-009-0025-5.

[ggge21000-bib-0044] Holbourn, A. , W. Kuhnt , M. Schulz , J.‐A. Flores , and N. Andersen (2007), Orbitally‐paced climate evolution during the middle Miocene “Monterey” carbon‐isotope excursion, Earth Planet. Sci. Lett., 261(3‐4), 534–550, doi:10.1016/j.epsl.2007.07.026.

[ggge21000-bib-0046] Holbourn, A. , W. Kuhnt , K. G. D. Kochhann , N. Andersen , and K. J. S. Meier (2015), Global perturbation of the carbon cycle at the onset of the Miocene Climatic Optimum, Geology, 43(2), 123–126, doi:10.1130/G36317.1.

[ggge21000-bib-0047] Hubert, B. , J. J. Álvaro , and J.‐Y. Chen (2005), Microbially mediated phosphatization in the Neoproterozoic Doushantuo Lagerstätte, South China, Bull. Soc. Geol. France, 176(4), 355–361, doi:10.2113/176.4.355.

[ggge21000-bib-0048] Jacobs, E. , H. Weissert , G. Shields , and P. Stille (1996), The Monterey Event in the Mediterranean: A record from shelf sediments of Malta, Paleoceanography, 11(6), 717–728, doi:10.1029/96PA02230.

[ggge21000-bib-0049] Jochum, K. P. et al. (2006), MPI‐DING reference glasses for in situ microanalysis: New reference values for element concentrations and isotope ratios, Geochem. Geophys. Geosyst., 7, Q02008, doi:10.1029/2005GC001060.

[ggge21000-bib-0050] Jochum, K. P. et al. (2011), Determination of Reference Values for NIST SRM 610–617 Glasses Following ISO Guidelines, Geostand. Geoanal. Res., 35(4), 397–429, doi:10.1111/j.1751-908X.2011.00120.x.

[ggge21000-bib-0051] Jochum, K. P. , D. Scholz , B. Stoll , U. Weis , S. A. Wilson , Q. Yang , A. Schwalb , N. Börner , D. E. Jacob , and M. O. Andreae (2012), Accurate trace element analysis of speleothems and biogenic calcium carbonates by LA‐ICP‐MS, Chem. Geol., 318–319, 31–44, doi:10.1016/j.chemgeo.2012.05.009.

[ggge21000-bib-0052] John, C. M. , K. B. Föllmi , E. de Kaenel , T. Adatte , P. Steinmann , and C. Badertscher (2002), Carbonaceous and phosphate‐rich sediments of the Miocene Monterey formation at El Capitan State Beach, California, USA, J. Sediment. Res., 72(2), 252–267.

[ggge21000-bib-0053] Karami, M. P. , A. De Leeuw , W. Krijgsman , P. T. Meijer , and M. J. R. Wortel (2011), The role of gateways in the evolution of temperature and salinity of semi‐enclosed basins: An oceanic box model for the Miocene Mediterranean Sea and Paratethys, Global Planet. Change, 79(1–2), 73–88, doi:10.1016/j.gloplacha.2011.07.011.

[ggge21000-bib-0054] Klinkhammer, G. P. , and M. R. Palmer (1991), Uranium in the oceans: Where it goes and why, Geochim. Cosmochim. Acta, 55(7), 1799–1806, doi:10.1016/0016-7037(91)90024-Y.

[ggge21000-bib-0055] Koenig, A. E. , R. R. Rogers , and C. N. Trueman (2009), Visualizing fossilization using laser ablation–inductively coupled plasma–mass spectrometry maps of trace elements in Late Cretaceous bones, Geology, 37(6), 511–514, doi:10.1130/G25551A.1.

[ggge21000-bib-0056] Kopp, R. E. , and J. L. Kirschvink (2008), The identification and biogeochemical interpretation of fossil magnetotactic bacteria, Earth Sci. Rev., 86(1–4), 42–61, doi:10.1016/j.earscirev.2007.08.001.

[ggge21000-bib-0057] Ku, T.‐L. , K. G. Knauss , and G. G. Mathieu (1977), Uranium in open ocean: Concentration and isotopic composition, Deep Sea Res., 24(11), 1005–1017, doi:10.1016/0146-6291(77)90571-9.

[ggge21000-bib-0058] Ter Kuile, B. , and J. Erez (1987), Uptake of inorganic carbon and internal carbon cycling in symbiont‐bearing benthonic foraminifera, Mar. Biol., 94(4), 499–509–509, doi:10.1007/BF00431396.

[ggge21000-bib-0059] Ter Kuile, B. , and J. Erez (1988), The size and function of the internal inorganic carbon pool of the foraminifer Amphistegina lobifera, Mar. Biol., 99(4), 481–487–487, doi:10.1007/BF00392555.

[ggge21000-bib-0060] Maloof, A. C. , R. E. Kopp , J. P. Grotzinger , D. A. Fike , T. Bosak , H. Vali , P. M. Poussart , B. P. Weiss , and J. L. Kirschvink (2007), Sedimentary iron cycling and the origin and preservation of magnetization in platform carbonate muds, Andros Island, Bahamas, Earth Planet. Sci. Lett., 259(3–4), 581–598, doi:10.1016/j.epsl.2007.05.021.

[ggge21000-bib-0061] McLennan, S. M. (1989), Rare earth elements in sedimentary rocks; Influence of provenance and sedimentary processes, Rev. Mineral. Geochem., 21(1), 169–200.

[ggge21000-bib-0062] McManus, J. , W. M. Berelson , G. P. Klinkhammer , D. E. Hammond , and C. Holm (2005), Authigenic uranium: Relationship to oxygen penetration depth and organic carbon rain, Geochim. Cosmochim. Acta, 69(1), 95–108, doi:10.1016/j.gca.2004.06.023.

[ggge21000-bib-0063] McNeill, D. F. (1990), Biogenic magnetite from surface holocene carbonate sediments, Great Bahama Bank, J. Geophys. Res., 95(B4), 4363–4371, doi:10.1029/JB095iB04p04363.

[ggge21000-bib-0064] Morad, S. , and S. Felitsyn (2001), Identification of primary Ce‐anomaly signatures in fossil biogenic apatite: Implication for the Cambrian oceanic anoxia and phosphogenesis, Sediment. Geol., 143(3–4), 259–264, doi:10.1016/S0037-0738(01)00093-8.

[ggge21000-bib-0065] Mourik, A. A. , H. A. Abels , F. J. Hilgen , A. Di Stefano , and W. J. Zachariasse (2011), Improved astronomical age constraints for the middle Miocene climate transition based on high‐resolution stable isotope records from the central Mediterranean Maltese Islands, Paleoceanography, 26, PA1210, doi:10.1029/2010PA001981.

[ggge21000-bib-0066] Mutti, M. , and D. Bernoulli (2003), Early marine lithification and hardground development on a Miocene ramp (Maiella, Italy): Key surfaces to track changes in trophic resources in nontropical carbonate settings, J. Sediment. Res., 73(2), 296–308, doi:10.1306/083102730296.

[ggge21000-bib-0067] Mutti, M. , D. Bernoulli , and P. Stille (1997), Temperate carbonate platform drowning linked to Miocene oceanographic events: Maiella platform margin, Italy, Terra Nova, 9(3), 122–125, doi:10.1046/j.1365-3121.

[ggge21000-bib-0070] O'Brien, G. W. , J. R. Harris , A. R. Milnes , and H. H. Veeh (1981), Bacterial origin of East Australian continental margin phosphorites, Nature, 294(5840), 442–444, doi:10.1038/294442a0.

[ggge21000-bib-0071] Patacca, E. , P. Scandone , and P. Mazza (2008), Oligocene migration path for Apulia macromammals: The central Adriatic bridge, Boll. Soc. Geol. Ital., 127(3), 335–337.

[ggge21000-bib-0072] Paytan, A. , and K. McLaughlin (2007), The oceanic phosphorus cycle, Chem. Rev., 107(2), 563–576, doi:10.1021/cr0503613. 17256993 10.1021/cr0503613

[ggge21000-bib-0074] Piper, D. Z. , and S. E. Calvert (2009), A marine biogeochemical perspective on black shale deposition, Earth Sci. Rev., 95(1‐2), 63–96, doi:10.1016/j.earscirev.2009.03.001.

[ggge21000-bib-0075] Reuter, M. , W. E. Piller , M. Harzhauser , O. Mandic , B. Berning , F. Rögl , A. Kroh , M. P. Aubry , U. Wielandt‐Schuster , and A. Hamedani (2009), The Oligo‐/Miocene Qom Formation (Iran): Evidence for an early Burdigalian restriction of the Tethyan Seaway and closure of its Iranian gateways, Int. J. Earth. Sci. (Geol. Rundsch.), 98(3), 627–650–650, doi:10.1007/s00531-007-0269-9.

[ggge21000-bib-0076] Reuter, M. , W. E. Piller , M. Brandano , and M. Harzhauser (2013), Correlating Mediterranean shallow water deposits with global Oligocene–Miocene stratigraphy and oceanic events, Global Planet. Change, 111, 226–236, doi:10.1016/j.gloplacha.2013.09.018. 10.1016/j.gloplacha.2013.09.018 PMC4376058 25844021

[ggge21000-bib-0077] Reynard, B. , C. Lécuyer , and P. Grandjean (1999), Crystal‐chemical controls on rare‐earth element concentrations in fossil biogenic apatites and implications for paleoenvironmental reconstructions, Chem. Geol., 155(3–4), 233–241, doi:10.1016/S0009-2541(98)00169-7.

[ggge21000-bib-0078] Rögl, F. (2000), Mediterranean and Paratethys. Facts and hypotheses of an Oligocene to Miocene paleogeography (short overview), Geol. Carpathica, 50(4), 339–349.

[ggge21000-bib-0079] Russell, A. D. , S. Emerson , B. K. Nelson , J. Erez , and D. W. Lea (1994), Uranium in foraminiferal calcite as a recorder of seawater uranium concentrations, Geochim. Cosmochim. Acta, 58(2), 671–681, doi:10.1016/0016-7037(94)90497-9.

[ggge21000-bib-0080] Ruttenberg, K. C. , and R. A. Berner (1993), Authigenic apatite formation and burial in sediments from non‐upwelling, continental margin environments, Geochim. Cosmochim. Acta, 57(5), 991–1007, doi:10.1016/0016-7037(93)90035-U.

[ggge21000-bib-0081] Schenau, S. J. , C. P. Slomp , and G. J. De Lange (2000), Phosphogenesis and active phosphorite formation in sediments from the Arabian Sea oxygen minimum zone, Mar. Geol., 169(1–2), 1–20, doi:10.1016/S0025-3227(00)00083-9.

[ggge21000-bib-0082] Shevenell, A. E. , J. P. Kennett , and D. W. Lea (2008), Middle Miocene ice sheet dynamics, deep‐sea temperatures, and carbon cycling: A Southern Ocean perspective, Geochem. Geophys. Geosyst., 9, Q02006, doi:10.1029/2007GC001736.

[ggge21000-bib-0083] Shields, G. , and P. Stille (2001), Diagenetic constraints on the use of cerium anomalies as palaeoseawater redox proxies: An isotopic and REE study of Cambrian phosphorites, Chem. Geol., 175(1‐2), 29–48, doi:10.1016/S0009-2541(00)00362-4.

[ggge21000-bib-0084] Slomp, C. P. , and P. Van Cappellen (2007), The global marine phosphorus cycle: Sensitivity to oceanic circulation, Biogeosciences, 4(2), 155–171, doi:10.5194/bg-4-155-2007.

[ggge21000-bib-0085] Slomp, C. P. , J. Thomson , and G. J. de Lange (2002), Enhanced regeneration of phosphorus during formation of the most recent eastern Mediterranean sapropel (S1), Geochim. Cosmochim. Acta, 66(7), 1171–1184, doi:10.1016/S0016-7037(01)00848-1.

[ggge21000-bib-0086] Spirakis, C. S. (1996), The roles of organic matter in the formation of uranium deposits in sedimentary rocks, Organ. Ore Depos., 11(1–3), 53–69, doi:10.1016/0169-1368(95)00015-1.

[ggge21000-bib-0087] Stamatakis, M. G. (2004), Phosphate deposits of Neogene age in Greece. Mineralogy, geochemistry and genetic implications, Chem. Erde Geochem., 64(4), 329–357.

[ggge21000-bib-0088] Steininger, F. F. , and G. Wessely (2000), From the Tethyan Ocean to the Paratethys Sea: Oligocene to Neogene stratigraphy, paleogeography and paleobiogeography of the circum‐Mediterranean region and the Oligocene to Neogene basin evolution in Austria, Mitt. Österr. Geol. Ges., 92(1999), 95–116.

[ggge21000-bib-0089] Tian, J. , A. Shevenell , P. Wang , Q. Zhao , Q. Li , and X. Cheng (2009), Reorganization of Pacific Deep Waters linked to middle Miocene Antarctic cryosphere expansion: A perspective from the South China Sea, Palaeogeogr. Palaeoclimatol. Palaeoecol., 284(3–4), 375–382, doi:10.1016/j.palaeo.2009.10.019.

[ggge21000-bib-0090] Tian, J. , M. Yang , M. W. Lyle , R. Wilkens , and J. K. Shackford (2013), Obliquity and long eccentricity pacing of the Middle Miocene climate transition, Geochem. Geophys. Geosyst., 14, 1740–1755, doi:10.1002/ggge.20108.

[ggge21000-bib-0091] van der Zee, C. , C. P. Slomp , and W. van Raaphorst (2002), Authigenic P formation and reactive P burial in sediments of the Nazaré canyon on the Iberian margin (NE Atlantic), Mar. Geol., 185(3–4), 379–392.

[ggge21000-bib-0092] Vecsei, A. , and D. G. K. Sanders (1999), Facies analysis and sequence stratigraphy of a Miocene warm‐temperate carbonate ramp, Montagna della Maiella, Italy, Sediment. Geol., 123(1–2), 103–127, doi:10.1016/S0037-0738(98)00079-7.

[ggge21000-bib-0093] Vecsei, A. , D. G. K. Sanders , D. Bernoulli , G. P. Eberli , and J. S. Pignatti (1998), Cretaceous to miocene sequence stratigraphy and evolution of the maiella carbonate platform margin, Italy, in Mesozoic and Cenozoic Sequence Stratigraphy of European Basins, SEPM Spec. Publ., vol. 60, edited by Graciansky P.‐C. et al., pp. 53–74, SEPM Soc. for Sediment. Geol, Tulsa, Olkahoma.

[ggge21000-bib-0094] Veeh, H. H. (1967), Deposition of uranium from the ocean, Earth Planet. Sci. Lett., 3, 145–150, doi:10.1016/0012-821X(67)90026-X.

[ggge21000-bib-0095] Veeh, H. H. , S. E. Calvert , and N. B. Price (1974), Accumulation of uranium in sediments and phosphorites on the South West African shelf, Mar. Chem., 2(3), 189–202, doi:10.1016/0304-4203(74)90014-0.

[ggge21000-bib-0096] Veizer, J. (1983), Trace elements and isotopes in sedimentary carbonates, Rev. Mineral. Geochem., 11, 265–299.

[ggge21000-bib-0097] Vincent, E. , and W. H. Berger (1985), Carbon dioxide and polar cooling in the Miocene: The Monterey hypothesis, Geophys. Monogr. Ser., 32, 455–468, doi:10.1029/GM032p0455.

[ggge21000-bib-0098] Williams, B. , J. Halfar , K. L. DeLong , S. Hetzinger , R. S. Steneck , and D. E. Jacob (2014), Multi‐specimen and multi‐site calibration of Aleutian coralline algal Mg/Ca to sea surface temperature, Geochim. Cosmochim. Acta, 139(0), 190–204, doi:10.1016/j.gca.2014.04.006.

[ggge21000-bib-0099] Webb, P. C. , M. Thompson , P. J. Potts , D. Long , B. Batjargal (2012), *GeoPT30: An International Proficiency Test for Analytical Geochemistry Laboratories: Report on Round 30 (Syenite, CG‐2) and 30A (Limestone, ML‐2)/January 2012* IAG (International association of Geoanalysts). [Available at http://www.geoanalyst.org/index.php/proficiency-testing-proficiency-testing/geopt-programme/previous-rounds.]

[ggge21000-bib-0100] Woodruff, F. , and S. Savin (1991), Mid‐Miocene isotope stratigraphy in the deep sea: High‐resolution correlations, paleoclimatic cycles, and sediment preservation, Paleoceanography, 6(6), 755–806, doi:10.1029/91pa02561.

[ggge21000-bib-0101] Woodruff, F. , and S. M. Savin (1989), Miocene deepwater oceanography, Paleoceanography, 4(1), 87–140, doi:10.1029/PA004i001p00087.

[ggge21000-bib-0102] Zachos, J. , M. Pagani , L. Sloan , E. Thomas , and K. Billups (2001a), Trends, rhythms, and aberrations in global climate 65 Ma to present, Science, 292(5517), 686–693, doi:10.1126/science.1059412. 11326091 10.1126/science.1059412

[ggge21000-bib-0103] Zachos, J. C. , N. J. Shackleton , J. S. Revenaugh , H. Pälike , and B. P. Flower (2001b), Climate response to orbital forcing across the oligocene‐miocene boundary, Science, 292(5515), 274–278, doi:10.1126/science.1058288. 11303100 10.1126/science.1058288

[ggge21000-bib-0104] Zachos, J. C. , G. R. Dickens , and R. E. Zeebe (2008), An early Cenozoic perspective on greenhouse warming and carbon‐cycle dynamics, Nature, 451(7176), 279–283, doi:10.1038/nature06588. 18202643 10.1038/nature06588

