# Supplementary material for: Orbitally paced phosphogenesis in Mediterranean shallow marine carbonates during the middle Miocene Monterey event
Source: Geochem Geophys Geosyst. 2016 Apr 29;17(4):1492–510. doi: 10.1002/2016GC006299 (PMC4984836; doi:10.1002/2016GC006299)
Supplement: Supplementary file 1 — Supporting Information S1 [file GGGE-17-1492-s001.doc]

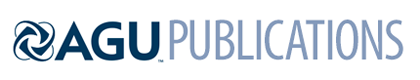


*Geochemistry, Geophysics, Geosystems*

Supporting Information for

**Orbitally paced phosphogenesis in Mediterranean shallow marine carbonates during the middle Miocene Monterey event**

Gerald Auer^1^, Christoph Hauzenberger^1^, Markus Reuter^1^, Werner E. Piller^1^

Institute for Earth Sciences, University of Graz, NAWI Graz, Heinrichstrasse 26, 8010 Graz, Austria.

**Additional Supporting Information (Files uploaded separately)**

Captions for Tables S1 to S5

**Introduction**

- Tables providing additional information of the LA-ICP-MS and EMP EDX analysis are provided as a separate Microsoft Excel® file using different spread sheets to better visualize the datasets.
- The file provides the principal results of: (1) The LA-ICP-MS analysis (see main text for information on were and how it was gathered); (2) Reproducibility (rel. error in %) of the standards measured as unknown (NIST 612 and MAPS-4); (3) Shale Normalization tables for the used REE elements, (4) EMP EDX results of the analyzed phosphate grains.

Table S1. LA-ICP-MS results after data reduction using Glitter (ver. 4.0).

Table S2. Table showing the reproducibility (deviation given in rel. % error) for the LA-ICP-MS analysis for the NIST 612 and MAPS-4 standards, that were measured as unknowns.

Table S3. Shale normalization for the measured REE elements using the World Shale Average (calculated just for reference) and the Post Archean Australian Shale (used for interpretation) normalization standards.

Table S4. Results of the EMP-EDX analysis of the investigated phosphates and carbonates.

Table S5. Results of the XRF bulk-samples together with the average gamma-ray intensity in recorded in the sample interval.
